# Corneal Thickness Profile and Associations in Chinese Children Aged 7 to 15 Years Old

**DOI:** 10.1371/journal.pone.0146847

**Published:** 2016-01-11

**Authors:** Yingyan Ma, Xiaofeng Zhu, Xiangui He, Lina Lu, Jianfeng Zhu, Haidong Zou

**Affiliations:** 1 Shanghai Eye Hospital, Shanghai Eye Disease Prevention & Treatment Center, Shanghai, China; 2 Department of Ophthalmology, Shanghai General Hospital, Shanghai Jiao Tong University School of Medicine, Shanghai, China; 3 Department of Maternal and Child Health, School of Public Health, Key Laboratory of Public Health Safety, Ministry of Education, Fudan University, Shanghai, China; Medical College of Soochow University, CHINA

## Abstract

Corneal thickness (CT) maps of the central (2-mm diameter), para-central (2 to 5-mm diameter), peripheral (5 to 6-mm diameter), and minimum (5-mm diameter) cornea were measured in normal Chinese school children aged 7 to 15 years old using Fourier-domain optical coherence tomography. Multiple regression analyses were performed to explore the effect of associated factors [age, gender, refraction, axial length and corneal curvature radius (CCR)] on CT and the relationship between central corneal thickness (CCT) and intraocular pressure (IOP). A total of 1228 eyes from 614 children were analyzed. The average CCT was 532.96 ± 28.33 μm for right eyes and 532.70 ±28.45 μm for left eyes. With a 10 μm increase in CCT, the IOP was elevated by 0.37 mm Hg, as measured by noncontact tonometry. The CT increased gradually from the center to the periphery. The superior and superior nasal regions had the thickest CTs, while the thinnest points were primarily located in the inferior temporal cornea. The CCT was associated with CCR (p = 0.008) but not with gender (p = 0.075), age (p = 0.286), axial length (p = 0.405), or refraction (p = 0.985). In the para-central region and the peripheral cornea, increased CT was associated with younger age, male gender, and a flatter cornea.

## Introduction

Corneal thickness (CT) is an important parameter for understanding corneal morphology and for the diagnosis and prognosis of many ophthalmic diseases in children and adults. Measuring normal CT values in the center of the cornea or in more peripheral areas could be beneficial in clinical practice. For the diagnosis of glaucoma, central corneal thickness (CCT) is considered an important parameter because the intraocular pressure is often overestimated in eyes with a thicker cornea [1]. In addition, eyes with a lesser CCT are at a higher risk of developing glaucoma; moreover, for glaucoma patients, a lesser CCT is a predictor of advanced glaucoma damage [2,3]. Children with congenital diseases such as Down syndrome exhibit a thinner CCT than healthy children, and a decreased CCT might be an early sign of degenerative corneal disease in these children [4]. In contrast, individuals with Turner syndrome present a thicker CCT compared with normal children [5]. Pachymetry maps involving para-central and peripheral cornea are also worthwhile for the diagnosis and monitoring of corneal diseases such as keratoconus, in which larger corneal asymmetry is present than in normal eyes [6]. School-age children are susceptible to various types of refractive errors. For those who wear contact lenses (i.e., rigid gas permeable contact lenses or orthokeratology contact lenses) to correct visual acuity, measuring both central and peripheral CT could provide a reference to assess changes in the cornea during treatment. For children with severe anisometropia or isoametropia who are unable to tolerate treatment with spectacles or contact lenses, refractive surgery could be performed as an alternative [7]; therefore, total corneal pachymetry maps along with knowledge of the position of the thinnest point provide valuable references for the selection of proper candidates for corneal refractive surgery by ophthalmic surgeons and for surgical design.

Few studies have reported CT maps for children, and even fewer exist for children of Chinese origin. Until now, only the Singapore Cohort Study of the Risk Factors for Myopia (SCORM), the Guangzhou Twin Eye Study, the Jinan City Eye Study, and the Xichang Pediatric Refractive Error Study have reported CT measurements for normal Chinese children with a relatively large sample size [8–11]. Most of those studies only measured CT within the central cornea. The Guangzhou Twin Eye Study provided a corneal point estimation for CT at 3 mm superior, inferior, nasal, and temporal to the pupil center; however, the participants included represented a special genetic cohort (twins). Thus far, no study has reported peripheral CT in normal Chinese children. In addition, the refraction or corneal curvature radius, which could be important factors associated with CT [8,12], were not included in previous studies of Chinese children.

FD-OCT is a high-speed, non-contact, 3-dimentional, high-resolution OCT. A corneal adaptor module is capable of generating a corneal pachymetry map with high repeatability and reproducibility compared with the gold standard of ultrasound pachymetry for both normal and keratoconus patients [13,14]. This study presents the CCT (2-mm diameter) as well as para-central (2 to 5-mm diameter) and peripheral (6-mm diameter) CT in normal Chinese schoolchildren using FD-OCT. The thickness and position of the thinnest point of the cornea were also demonstrated. In addition, regression analyses were performed for central, para-central, peripheral, and the thinnest CT to explore possible associated factors.

## Methods

One primary school and one middle school in Shanghai, China were randomly selected, and students with odd student code numbers were included in the study. Inclusion criteria were that the children were of Chinese nationality and aged 7 to 15 years old. Exclusion criteria were that the children had ocular diseases with the exception of refractive error, or that the children had a history of wearing soft or hard contact lenses (in the last 4 weeks). Children who were uncooperative during examinations or who were without written informed consent from their parents or guardians were also excluded. To achieve a 95% confidence interval wherein the mean fell within the range of one-third of the expected standard deviation, the sample size required was 35 for each age interval [15].

The study adhered to the tenets of the Declaration of Helsinki and was approved by the Ethics Committee of Shanghai General Hospital, Shanghai Jiao Tong University. The examinations were conducted during weekdays while the students were in class in May and June 2014. One ophthalmologist, three optometrists, and two public health doctors participated in the examinations. The children first underwent a basic eye examination that included a test of uncorrected visual acuity (Standard Logarithmic Visual Acuity E Chart, 5 m) and a slit lamp examination. Corneal thickness measurements were then carried out by RTVue Fourier optical coherence tomography (FD-OCT) (Optovue, Inc, Fremont, CA, USA) with a wide-angle (long) corneal adapter module lens (CAM) in the “pachymetry” scan pattern (6-mm diameter scan, 8 meridians, 1024 axial-scans each, 5 times repeated). Each child’s head was stabilized with a chin rest, and they were asked to stare at an internal fixation target in the OCT. The pachymetry scans were centered at the pupil, and the images were displayed on the screen in real time to aid with alignment. If the children rotated their eyes or blinked during the measurement, another scan was taken to ensure quality. To measure the test-retest repeatability of the FD-OCT for corneal thickness, the first 25 students from each school were selected. The intraclass correlation coefficient values between the two measurements were 0.991 for the central corneal thickness.

**Fig 1:**
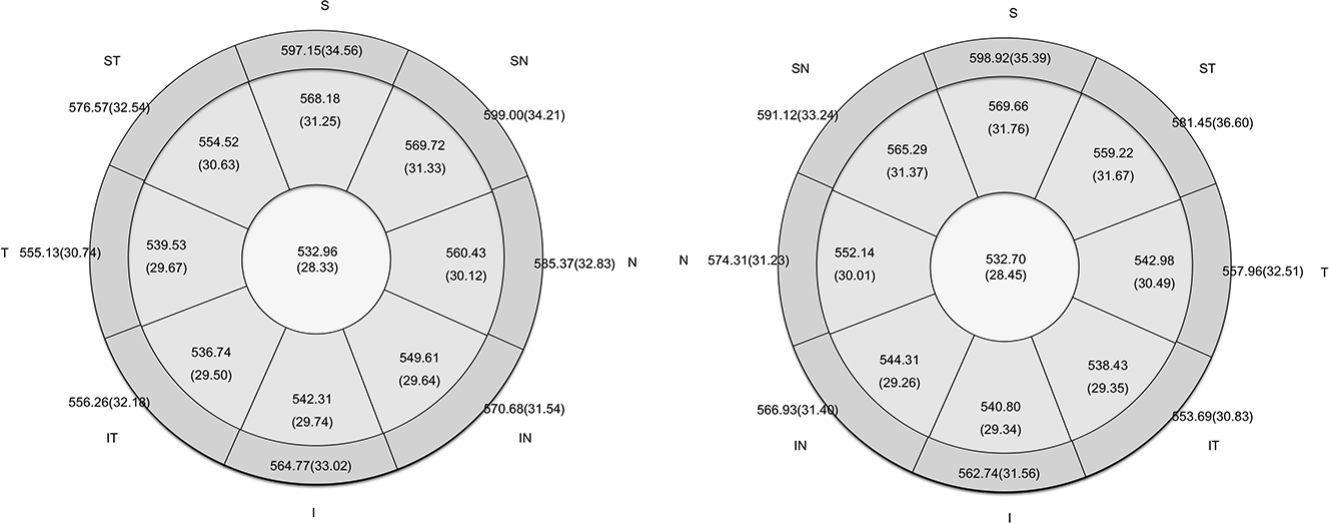
Corneal Thickness Measured by RTVue Fourier Optical Coherence Tomography for 614 Chinese Children (Mean (SD)). The average corneal thickness (SD) for 614 Chinese Children in the left eye (Left) and the right eye (Right). S: Superior, ST: Superior Temporal, ST: Temporal, IT: Inferior Temporal, I: Inferior, IN: Inferior Nasal, N: Nasal, SN: Superior Nasal. SD: Standard Deviation.

RTVue-CAM software (version 6.11) automatically processed the OCT scan to generate a pachymetry map of corneal thickness (within a 6-mm diameter range), including the minimum thickness and its position (within a 5-mm diameter range and marked with an asterisk). The maps were divided into 17 sectors: one 2-mm diameter pupil center (C), eight 2 to 5 mm para-central sectors [superior (S), superotemporal (ST), temporal (T), inferotemporal (IT), inferior (I), inferonasal (IN), nasal (N), superonasal (SN)] and eight 5 to 6-mm diameter peripheral sectors (S, ST, T, IT, I, IN, N, SN) (Fig 1). The average thickness of each sector was calculated and displayed automatically in the corresponding regions for CT.

After the measurement of corneal thickness, intraocular pressure (IOP) was recorded using a noncontact tonometer (T-1000, Nidek, Japan) because this method was reported to achieve a much higher success rate than eye-contact Goldmann applanation tonometry for measuring IOP in children younger than 10 years old [16]. Children with a normal IOP (between 12 to 22 mm Hg) and with written informed consent for cycloplegia were given 1% cyclopentolate (Cyclogyl; Alcon, Fort Worth, TX, USA) to dilate the pupil, and the corneal curvature radius was measured using an autorefractor (KR-8800, Topcon, Tokyo), the axial length was measured by IOLMaster (version 5.02, Carl Zeiss Meditec, Oberkochen, Germany), and the best-corrected visual acuity was determined if the uncorrected visual acuity was lower than 20/25 in either eye. A detailed explanation of all cycloplegia procedures has been provided in a previous study [17].

A one-sample Kolmogorov-Smirnov Z test was used to evaluate the normalcy of the corneal thickness distribution. To compare the differences between the right and left eyes, a paired sample t-test was used for normally distributed data, and a Wilcoxon signed-rank test was used for non-normally distributed data. For evaluating the correlations of CT between the right and left eyes, the Pearson correlation coefficient was used. Multiple linear regressions were performed to explore the effect of gender (i.e., girls vs boys), age, central corneal thickness (CCT), axial length, refraction (spherical equivalent = spherical degree + 0.5*cylindrical degree), and corneal curvature radius with intraocular pressure. To explore the possible factors associated with CT, gender (i.e., girls vs boys), age, axial length, refraction and corneal curvature radius were included in the regression analyses by stepwise mode. P values of less than 0.05 were considered statistically significant. SPSS 16.0 (SPSS Institute, Inc., Chicago, IL) was used for all statistical analyses.

## Results

**Fig 2:**
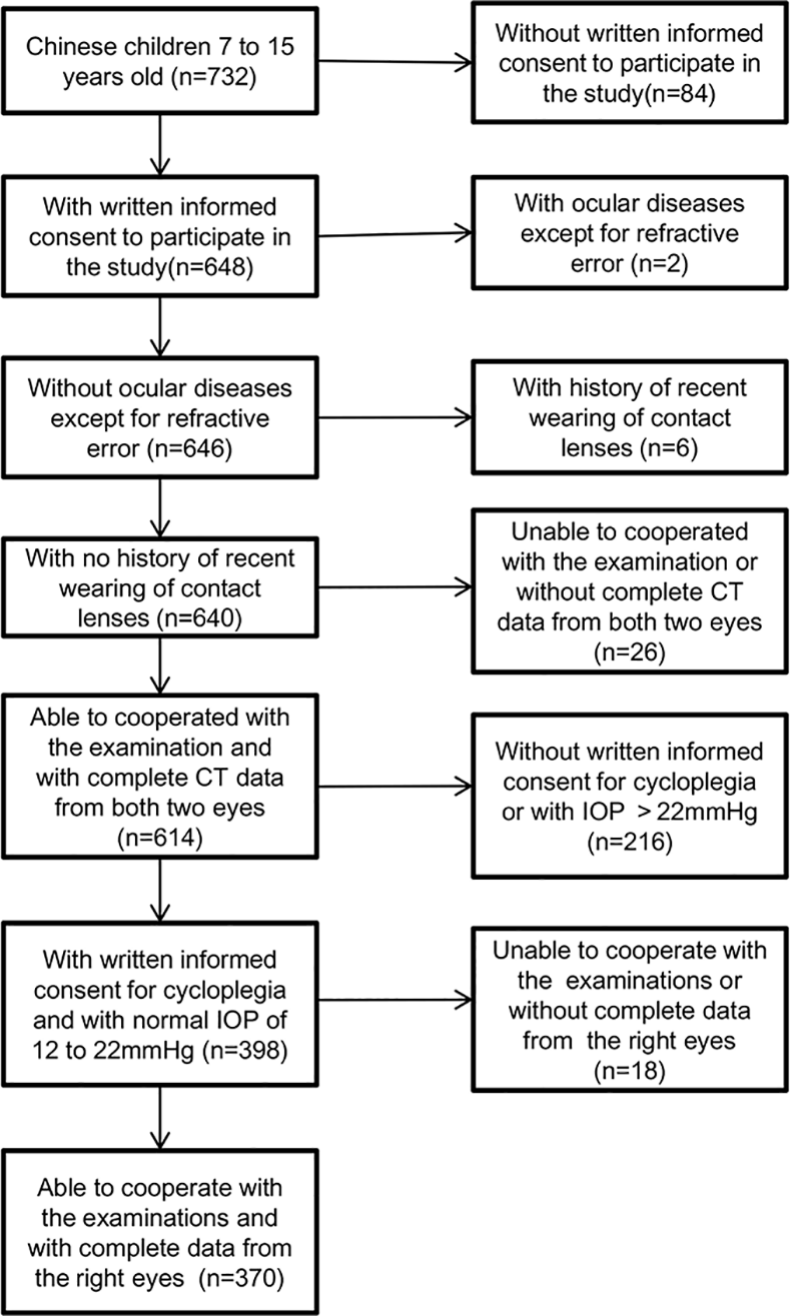
Inclusion and exclusion flowchart for analyzing corneal thickness and associated factors. CT, corneal thickness; IOP, intraocular pressure.

Among the 732 students who met the inclusion criteria, 648 children agreed to participate in the study, and CTs of 1228 eyes from 614 children were included in the analyses. Detailed inclusion and exclusion procedures are described in the flowchart in Fig 2. Among those included in the analyses, 312 (50.8%) children were male. The mean age was 11.24 (SD = 2.34).

**Fig 3:**
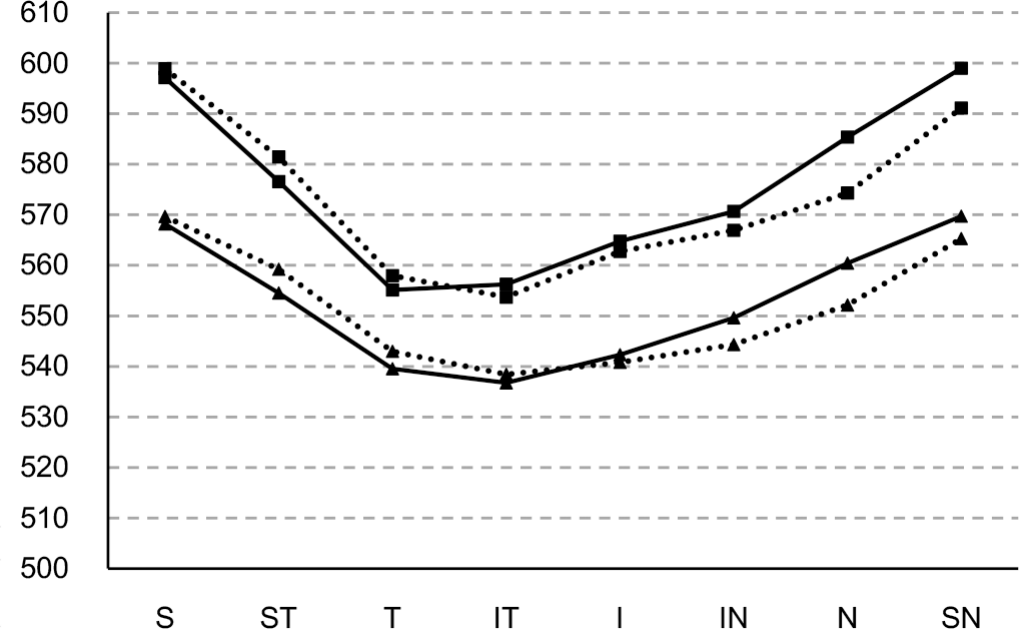
Para-central (2 to 5 mm) and peripheral (5 to 6 mm) corneal thickness in 614 Chinese children. Full line with triangle: CT from the right eye in 2 to 5-mm diameter regions; full line with square: CT from the right eye in 5 to 6-mm diameter regions; dotted line with triangle: CT from the left eye in 2 to 5-mm diameter regions; dotted line with square: CT from the right eye in 5 to 6-mm diameter regions. S: Superior, ST: Superior Temporal, ST: Temporal, IT: Inferior Temporal, I: Inferior, IN: Inferior Nasal, N: Nasal, SN: Superior Nasal.

Table 1 and Fig 1 summarize the corneal thickness in various positions for both eye sides. In the center area, corneal thickness did not differ significantly between the right and left eyes (p = 0.369). In the para-central and peripheral areas, corneal thickness was different between the right and left eyes (Fig 3, Table 1), although the correlation coefficients between them were relatively high for all positions (Table 1). The central corneal thickness was thinner than the peripheral corneal thickness in areas 2 to 5 mm from the center of pupil; corneal thickness in areas 2 to 5 mm from the center of pupil was thinner than in areas 6 mm from the center (Fig 3).

**Table 1:** Corneal thickness in the central (2-mm diameter), para-central (2 to 5-mm diameter), and peripheral (5 to 6-mm diameter) cornea in both eyes of 614 normal Chinese children.

	Right Eye		Left Eye		Correlation Coefficient^a^	Paired t Test
	Mean (μm)	SD	Mean (μm)	SD		P value
**Center**	532.96	28.33	532.70	28.45	0.97	0.369
**Superior (2–5 mm)**	568.18	31.25	569.66	31.76	0.86	0.027
**Superior (6 mm)**	597.15	34.56	598.92	35.39	0.75	0.079
**Superior Temporal (2–5 mm)**	554.52	30.63	559.22	31.67	0.86	<0.001
**Superior Temporal (6 mm)**	576.57	32.54	581.45	36.60	0.77	<0.001
**Temporal (2–5 mm)**	539.53	29.67	542.98	30.49	0.89	<0.001
**Temporal (6 mm)**	555.13	30.74	557.96	32.51	0.85	<0.001
**Inferior Temporal (2–5 mm)**	536.74	29.50	538.43	29.35	0.94	<0.001
**Inferior Temporal (6 mm)**	556.26	32.18	553.69	30.83	0.85	<0.001
**Inferior (2–5 mm)**	542.31	29.74	540.80	29.34	0.94	<0.001
**Inferior (6 mm)**	564.77	33.02	562.74	31.56	0.85	0.005
**Inferior Nasal (2–5 mm)**	549.61	29.64	544.31	29.26	0.92	<0.001
**Inferior Nasal (6 mm)**	570.68	31.54	566.93	31.40	0.86	<0.001
**Nasal (2–5 mm)**	560.43	30.12	552.14	30.01	0.87	<0.001
**Nasal (6 mm)**	585.37	32.83	574.31	31.23	0.82	<0.001
**Superior Nasal (2–5 mm)**	569.72	31.33	565.29	31.37	0.84	<0.001
**Superior Nasal (6 mm)**	599.00	34.21	591.12	33.24	0.74	<0.001

SD = standard deviation.

a. Pearson correlation coefficient, all P<0.01

**Fig 4:**
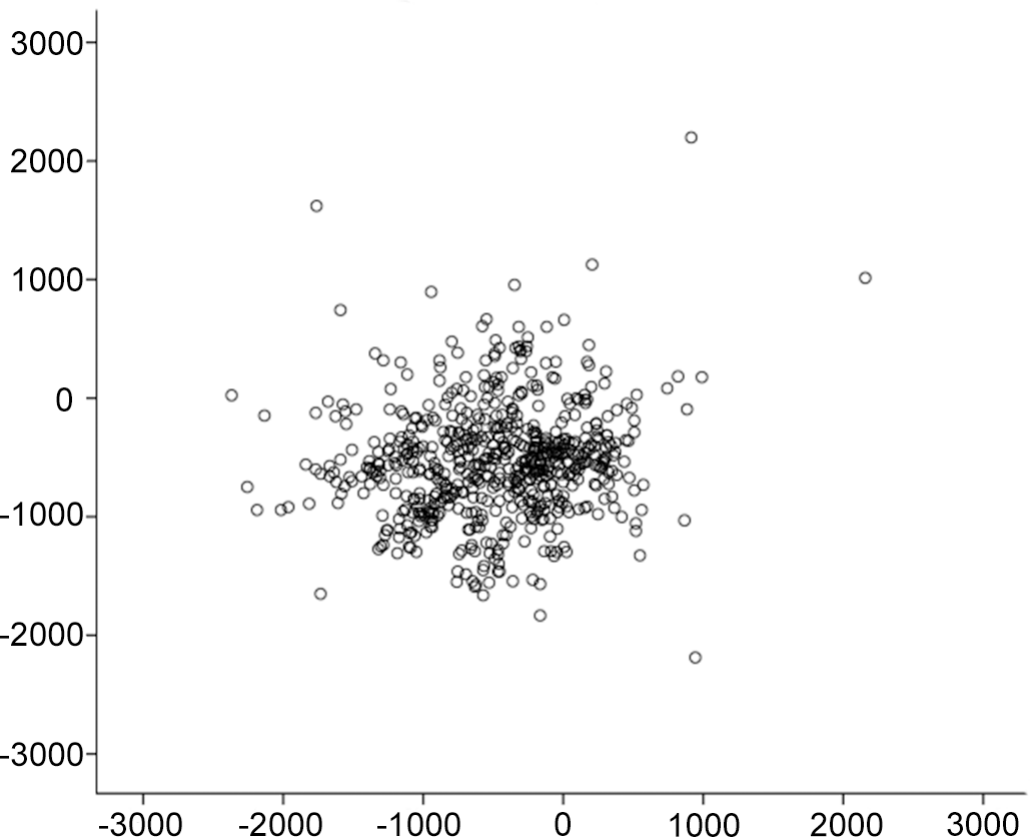
Scatter plot indicating the position of minimum corneal thickness in the area 5 mm from the pupil center in the right eye of 614 normal Chinese children. Along the vertical axis, a negative number represents the distance inferior to the pupil center, while a positive number represents the distance superior to the pupil center (μm). Along the horizontal axis, a negative number represents the distance temporal to the pupil center, while a positive number represents the distance nasal to the pupil center (μm).

**Fig 5:**
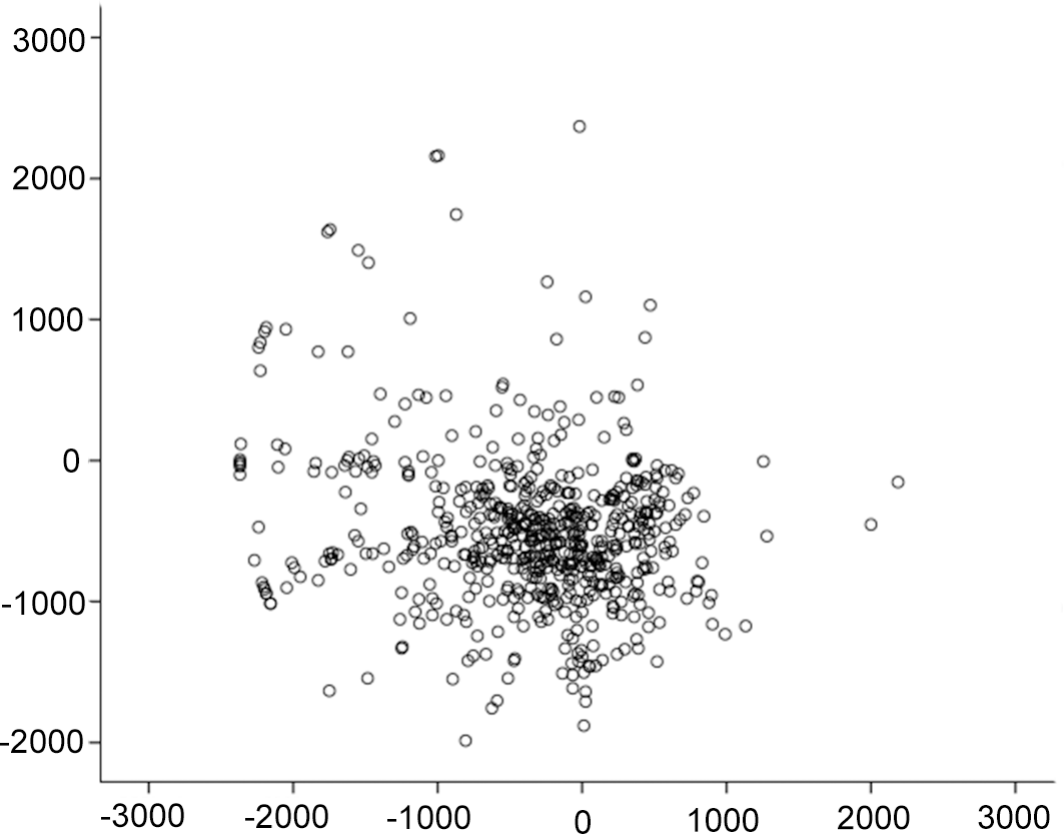
Scatter plot of the position of minimum corneal thickness in the left eyes of 614 normal Chinese children. Along the verticat axis, a negative number represents the distance inferior to the pupil center, while a positive number represents the distance superior to the pupil center (μm). Along the horizontal axis, a negative number represents the distance temporal to the pupil center, while a positive number represents the distance nasal to the pupil center (μm).

The minimum CT and its locations are summarized in Table 2. The CT of the thinnest point was significantly thinner than that of the pupil center in both the right and left eye (paired t-test, both p<0.001). The medium position of the minimum CT was located in the inferior temporal region of the cornea (Table 2, Figs 4 and 5). The CT of the minimum point differed significantly between the right and left eyes. The scatter plot for the location of the minimum CT in right eyes is presented in Fig 4 (a scatter plot for left eyes is presented in Fig 5).

**Table 2:** Values and locations of the minimum corneal thickness in the area 5 mm from the pupil center in 614 normal Chinese children.

	Right Eye	Left Eye	P value
**Minimum Corneal Thickness [Mean(SD), μm]**	523.35 (28.41)	521.49(29.19)	<0.001^c^
**Vertical Location [Median(Percentile), μm]**^a^	-548 (-833 to -294)	-557 (-819 to -248)	0.615^d^
**Horizontal Location [Median(Percentile), μm]**^b^	-454 (-884 to -94)	-248 (-707 to 124)	<0.001^e^

SD = standard deviation.

a. Negative numbers indicate the distance inferior to the pupil center, while positive numbers indicate the distance superior to the pupil center.

b. Negative numbers indicate the distance temporal to the pupil center, while positive numbers indicate the distance nasal to the pupil center.

c. Paired t test, t = 3.885

d. Wilcoxon signed ranks test, z = -0.503

e. Wilcoxon signed ranks test, z = -17.386

To explore putative factors correlated with CT and the relationship between ocular pressure and CT, 370 right eyes from 370 children with complete measurements for cycloplegic autorefraction, axial length, corneal radius, and intraocular pressure were utilized (Fig 2). The mean age was 11.61 (SD = 2.13), and 168 (45.41%) of the children were boys. The age was statistically older (student-t test, P<0.001), and the proportion of male children was significantly reduced (chi-square test, P = 0.001) in children who were included in subsequent analyses, probably because older children and girls are more cooperative in examinations than younger children and boys.

Multiple regression analysis indicated that for every 10 μm increase in CCT, the intraocular pressure was increased by 0.37 mmHg (p<0.001) after adjusting for sex. CCT was associated with the corneal curvature radius (CCR, p = 0.008) but not with sex (p = 0.075), age (p = 0.286), axial length (p = 0.405), or refractive error (p = 0.985). The minimum CT was also correlated with CCR (p = 0.001). In the inferior temporal and inferior areas (both 2 to 5 mm and 6-mm diameter), the CT was inversely associated with age. Males exhibited increased CTs in most regions of the cornea, including the superior, superior temporal, temporal, inferior temporal and inferior areas. CCR was positively correlated with the temporal, inferior temporal, inferior, inferior nasal, and nasal areas of the CT (Table 3). The CTs according to different age groups and genders were analyzed for the 614 children in the primary analyses and are listed in Tables 4 and 5; the CTs classified by quartile corneal curvature radius are presented in Table 6.

**Table 3:** Factors associated with corneal thickness in the central (2-mm diameter), para-central (2 to 5-mm diameter), and peripheral (5 to 6-mm diameter) cornea by multiple regression analyses (n = 370, data from right eyes).

Dependent Variable	Independent Variables Included in Equation	Beta Coefficient	95% CI of Beta Coefficient	P Value^a^
**Center**	CCR	14.88	3.84 to 25.91	0.008
**Superior (2–5 mm)**	Gender^b^	-6.53	-12.98 to -0.09	0.047
**Superior (6 mm)**	None	/	/	/
**Superior Temporal (2–5 mm)**	Gender^b^	-7.41	-13.73 to -1.08	0.022
**Superior Temporal (6 mm)**	Gender^b^	-7.70	-14.32 to -1.08	0.023
**Temporal (2–5 mm)**	CCR	16.57	4.80 to 28.33	0.006
**Temporal (6 mm)**	Gender^b^	-7.94	-14.38 to -1.49	0.016
**Inferior Temporal (2–5 mm)**	CCR	12.89	0.77 to 25.02	0.037
	Gender^b^	-7.74	-14.17 to -1.31	0.019
	Age	-1.54	-3.00 to -0.08	0.039
**Inferior Temporal (6 mm)**	CCR	11.76	-1.70 to 25.22	0.087
	Age	-2.10	-3.72 to -0.48	0.011
	Gender^b^	-8.06	-15.20 to -0.92	0.027
**Inferior (2–5 mm)**	CCR	13.81	1.70 to 25.92	0.026
	Gender^b^	-7.85	-14.28 to -1.43	0.017
	Age	-1.68	-3.14 to -0.23	0.024
**Inferior (6 mm)**	Age	-2.82	-4.44 to -1.21	0.001
	Gender^b^	-9.25	-16.38 to -2.12	0.011
	CCR	14.70	1.26 to 28.14	0.032
**Inferior Nasal (2–5 mm)**	CCR	17.60	6.04 to 29.15	0.003
**Inferior Nasal (6 mm)**	CCR	16.40	3.75 to 29.04	0.011
**Nasal (2–5 mm)**	CCR	14.04	2.34 to 25.75	0.019
**Nasal (6 mm)**	None	/	/	/
**Superior Nasal (2–5 mm)**	None	/	/	/
**Superior Nasal (6 mm)**	None	/	/	/

CCR = corneal curvature radius; CI = confidence interval.

a. Linear multiple regression analysis, step-wise mode

b. Girls vs boys

**Table 4:** Corneal thickness in the entral (2-mm diameter), para-central (2 to 5-mm diameter), and peripheral (5 to 6-mm diameter) areas according to age interval in 614 normal Chinese children.

	Age (years old)																			
	7		8		9		10		11		12		13		14		15		total	
	n = 54		n = 43		n = 63		n = 62		n = 86		n = 93		n = 88		n = 88		n = 37		n = 614	
	Mean	SD	Mean	SD	Mean	SD	Mean	SD	Mean	SD	Mean	SD	Mean	SD	Mean	SD	Mean	SD	Mean	SD
**C**	532.98	26.76	541.37	27.50	533.94	25.58	533.16	27.91	534.20	28.86	528.54	29.79	531.32	27.44	533.52	31.09	532.00	27.23	532.96	28.33
**S1**	562.65	27.65	572.81	28.33	567.37	27.85	566.79	32.32	570.14	32.68	565.61	34.81	567.38	29.59	571.28	33.58	571.08	29.47	568.18	31.25
**S2**	594.13	33.13	603.88	35.00	597.92	32.69	599.35	34.40	601.09	36.69	595.45	37.04	592.92	32.39	595.52	35.58	597.78	31.45	597.15	34.56
**ST1**	550.81	27.90	558.86	28.86	552.17	27.43	551.89	31.00	556.14	31.48	552.73	34.34	554.14	29.24	557.59	32.24	557.65	29.79	554.52	30.63
**ST2**	569.31	28.94	578.09	33.50	573.83	29.92	573.26	32.53	578.76	34.17	575.27	35.98	577.60	30.68	580.08	33.19	583.03	30.79	576.57	32.54
**T1**	536.59	27.35	547.19	30.21	538.16	26.69	537.84	28.41	542.26	31.08	536.81	31.85	537.75	28.35	541.69	31.60	539.65	29.29	539.53	29.67
**T2**	549.35	28.75	560.65	31.12	552.46	28.09	552.56	29.51	555.49	29.58	554.49	33.98	555.15	29.30	559.01	33.50	557.51	30.82	555.13	30.74
**IT1**	537.26	30.18	547.63	30.96	538.03	28.02	538.37	27.05	537.26	28.39	532.61	30.12	533.97	28.21	536.52	32.24	534.70	29.92	536.74	29.50
**IT2**	555.63	34.48	568.81	34.54	558.00	33.13	560.56	30.20	557.45	29.85	552.77	32.32	551.72	30.07	554.84	33.71	552.51	31.98	556.26	32.18
**I1**	544.02	31.65	554.84	31.26	544.95	27.75	545.42	26.81	543.16	28.49	536.43	29.21	538.44	28.17	541.69	33.27	539.00	29.21	542.31	29.74
**I2**	567.80	37.38	582.51	36.41	569.41	32.87	573.23	30.83	566.63	30.20	558.59	31.31	556.69	29.20	560.63	34.90	557.97	30.41	564.77	33.02
**IN1**	550.63	30.24	560.72	30.00	553.32	27.46	551.87	28.57	550.52	28.81	543.09	29.07	546.97	28.54	549.20	33.64	546.70	27.22	549.61	29.64
**IN2**	572.28	33.13	581.07	37.93	576.33	29.54	575.63	28.69	571.48	29.02	563.49	29.54	566.25	30.63	569.28	35.77	568.38	27.40	570.68	31.54
**N1**	559.30	28.48	568.26	28.47	562.27	27.52	561.21	31.00	561.78	31.16	554.11	30.77	558.59	28.89	563.16	33.48	559.22	26.72	560.43	30.11
**N2**	580.48	33.18	590.02	29.71	585.21	29.07	584.85	34.40	586.91	33.90	581.13	32.42	584.27	32.91	589.42	36.69	588.27	28.40	585.37	32.83
**SN1**	567.80	28.82	576.44	29.08	570.02	28.10	570.02	33.09	571.33	32.86	565.81	33.27	568.01	29.65	571.10	34.57	570.68	27.85	569.72	31.32
**SN2**	596.63	34.76	609.26	35.10	601.87	31.42	602.85	36.15	601.99	35.61	595.18	35.12	594.38	32.50	596.82	35.27	598.08	28.18	599.00	34.21

C = Center 2 mm; S1 = Superior 2–5 mm; S2 = Superior 5–6 mm; ST1 = Superior Temporal 2–5 mm; ST2 = Superior 5–6 mm; ST1 = Temporal 2–5 mm; ST2 = Temporal 5–6 mm; IT1 = Inferior Temporal 2–5 mm; IT2 = Inferior Temporal 5–6 mm; I1 = Inferior 2–5 mm; I2 = Inferior 5–6 mm; IN1 = Inferior Nasal 2–5 mm; IN2 = Inferior Nasal 5–6 mm; N1 = Nasal 2–5 mm; N2 = Nasal 5–6 mm; SN1 = Superior Nasal 2–5 mm; SN2 = Superior Nasal 5–6 mm; SD = Standard Deviation.

**Table 5:** Corneal thickness in the central (2-mm diameter), para-central (2 to 5-mm diameter), and peripheral (5 to 6-mm diameter) areas, according to gender in 614 normal Chinese children.

	Gender					
	male (n = 312)		female (n = 302)		Total (n = 614)	
	Mean	SD	Mean	SD	Mean	SD
**C**	535.10	28.82	530.76	27.69	532.96	28.33
**S1**	569.39	31.52	566.93	30.98	568.18	31.25
**S2**	597.84	34.45	596.44	34.71	597.15	34.56
**ST1**	556.24	30.66	552.74	30.54	554.52	30.63
**ST2**	578.00	32.50	575.10	32.57	576.57	32.54
**T1**	541.63	29.36	537.35	29.89	539.53	29.67
**T2**	556.83	30.42	553.37	31.02	555.13	30.74
**IT1**	539.50	29.70	533.89	29.07	536.74	29.50
**IT2**	558.79	32.16	553.64	32.04	556.26	32.18
**I1**	545.35	30.43	539.17	28.73	542.31	29.74
**I2**	568.34	33.95	561.08	31.67	564.77	33.02
**IN1**	552.05	30.65	547.10	28.39	549.61	29.64
**IN2**	573.02	32.91	568.26	29.93	570.68	31.54
**N1**	562.07	30.92	558.74	29.22	560.43	30.11
**N2**	586.34	34.17	584.37	31.41	585.37	32.83
**SN1**	570.45	32.06	568.97	30.58	569.72	31.32
**SN2**	599.42	35.34	598.57	33.06	599.00	34.21

C = Center 2-mm; S1 = Superior 2-5mm; S2 = Superior 5-6mm; ST1 = Superior Temporal 2-5mm; ST2 = Superior 5-6mm; ST1 = Temporal 2-5mm; ST2 = Temporal 5-6mm; IT1 = Inferior Temporal 2-5mm; IT2 = Inferior Temporal 5-6mm; I1 = Inferior 2-5mm; I2 = Inferior 5-6mm; IN1 = Inferior Nasal 2-5mm; IN2 = Inferior Nasal 5-6mm; N1 = Nasal 2-5mm; N2 = Nasal 5-6mm; SN1 = Superior Nasal 2-5mm; SN2 = Superior Nasal 5-6mm; SD = Standard Deviation.

**Table 6:** Corneal thickness in the central (2-mm diameter), para-central (2 to 5-mm diameter), and peripheral (5 to 6-mm diameter) areas, according to the quartile corneal curvature radius in normal Chinese children (n = 370).

	Corneal Curvature Radius (mm)														
	7.15 to 7.65			7.66 to 7.83			7.84 to 8.00			8.01 to 8.60			Total		
	Mean	No.	SD	Mean	No.	SD	Mean	No.	SD	Mean	No.	SD	Mean	No.	SD
**C**	528.68	88	29.131	527.34	94	28.706	534.35	91	26.718	539.45	97	29.002	532.56	370	28.714
**S1**	566.43	88	32.065	564.68	94	31.866	569.00	91	29.470	573.56	97	32.236	568.49	370	31.496
**S2**	593.88	88	36.639	594.59	94	33.681	598.02	91	34.224	601.65	97	36.379	597.11	370	35.244
**ST1**	553.01	88	32.251	550.55	94	31.962	554.97	91	28.845	560.00	97	30.526	554.70	370	30.992
**ST2**	574.95	88	34.203	574.21	94	33.499	577.75	91	29.593	581.76	97	32.193	577.24	370	32.417
**T1**	535.41	88	31.493	533.68	94	30.702	540.00	91	28.439	547.43	97	30.463	539.25	370	30.647
**T2**	552.34	88	33.335	550.46	94	31.768	556.58	91	30.057	561.52	97	30.559	555.31	370	31.595
**IT1**	530.74	88	32.003	530.17	94	30.368	538.76	91	28.658	543.37	97	29.646	535.88	370	30.566
**IT2**	550.44	88	35.885	549.65	94	33.817	559.13	91	32.784	562.33	97	31.876	555.49	370	33.892
**I1**	535.80	88	31.710	535.00	94	29.563	544.78	91	28.859	548.70	97	30.496	541.19	370	30.613
**I2**	556.97	88	36.164	557.48	94	32.573	567.56	91	32.792	570.90	97	33.911	563.35	370	34.279
**IN1**	544.16	88	30.785	542.29	94	28.985	552.22	91	28.337	555.99	97	30.759	548.77	370	30.160
**IN2**	564.90	88	33.522	563.45	94	30.796	573.84	91	29.829	576.20	97	35.614	569.69	370	32.890
**N1**	557.27	88	30.153	554.45	94	29.981	561.99	91	28.092	567.36	97	31.983	560.36	370	30.401
**N2**	583.28	88	32.025	579.34	94	34.068	587.97	91	29.838	591.27	97	35.576	585.53	370	33.194
**SN1**	568.16	88	31.328	565.56	94	31.423	571.13	91	29.031	574.65	97	33.845	569.93	370	31.554
**SN2**	597.01	88	34.196	594.91	94	33.499	600.59	91	31.144	603.77	97	38.977	599.13	370	34.672

C = Center 2-mm; S1 = Superior 2-5mm; S2 = Superior 5-6mm; ST1 = Superior Temporal 2-5mm; ST2 = Superior 5-6mm; ST1 = Temporal 2-5mm; ST2 = Temporal 5-6mm; IT1 = Inferior Temporal 2-5mm; IT2 = Inferior Temporal 5-6mm; I1 = Inferior 2-5mm; I2 = Inferior 5-6mm; IN1 = Inferior Nasal 2-5mm; IN2 = Inferior Nasal 5-6mm; N1 = Nasal 2-5mm; N2 = Nasal 5-6mm; SN1 = Superior Nasal 2-5mm; SN2 = Superior Nasal 5-6mm; SD = Standard Deviation.

## Discussion

Recent studies have proposed that racial differences influence the results of CT measurement in children as well as adults [12], lending value to CT profiles from individuals of different ethnicities. CCT measurements varied from 523 to 579 μm in children from different countries or racial groups [12,18]; East-Asian children were reported to have a thinner CCT than white children and thicker cornea than African-American children [12], while Chinese children were reported to have a thicker CCT than Malayan or Indian children [8]. Our results were similar to the results reported among Chinese school-aged children; 550.7 ± 32.8 μm by Pentacam, 553 ±33 μm by ultrasound and 554.19 ± 35.46 μm by Tonopachy NT-530P, and in Singaporean schoolchildren, who were mostly of Chinese descent: 543.6 ± 32.0 μm by optical low-coherence reflectometry (LCR) pachymeter [8–11]. These differences might be explained by the instruments used for measurement; the thickness measured by FD-OCT was usually smaller than that measured by Pentacam or ultrasound pachymetry [14,19]. Our results were comparable to the mean CCT reported in normal adults measured using FD-OCT (532 μm in 66 eyes of subjects at a mean age of 35.39±10.6 years, and 537 μm in 561 eyes of subjects at a mean age of 45.70±21.20 years) [13,20].

Zheng and associates demonstrated that in 926 school-aged twin children, the CT 3 mm from the pupil center was thickest in the superior region (656.0 ± 38.7 μm) followed by the nasal region (642.1 ± 37.2 μm), the inferior region (627.9 ± 36.6 μm), and the temporal region (612.5 ± 36.3 μm) [9]. They also determined that the CT at the thinnest points was 533.2 ± 30.0 μm and was located mostly in the inferior temporal quadrant [9]. However, Hussein and associates reported that the CT at 3 mm was thickest in the superior region (575 ± 52 μm) followed by the temporal (574 ± 47 μm), the inferior (568 ± 51 μm), and the nasal (568 ± 50 μm) regions [21]. In our study, the FD-OCT automatically outputs para-central (i.e., the 2 mm to 5-mm diameter region) and peripheral (the 6-mm diameter region) CT data for 8 zones in the cornea. Consistent with established knowledge, the CT increased gradually from the central to the peripheral cornea [22,23]. The thickest regions were located in the superior nasal regions; the inferior temporal regions had the thinnest mean CT in the para-central and peripheral regions, which correspond to the median horizontal and vertical locations of the thinnest points (Table 2, Figs 4 and 5). Similar distributions of CT data were observed in studies performed in adults [22,24].

The CCT was obviously thicker than the thinnest CT in both eyes (approximately 10μm), and the medium positions of the thinnest points were located in the inferior temporal region, similar to previous values reported for both children and adults [23–26]. The thinnest CT was thicker in the right eye than in the left eye, and the medium horizontal positions of the thinnest points were located more temporally in right eyes than in left eyes. These laterality differences were also observed in other studies [23,27]. However, whether the thinnest CT was thicker in the right eye or in the left eye was inconsistent between studies, probably due to the different instruments used for measurement or the different racial populations included [23,27,28]. Because the thinnest point of the cornea was not located in the pupil’s center, measuring CCT alone might not be sufficient to identify refractive surgery candidates and to prepare for surgery. Furthermore, the thickness of the thinnest point was reported to be associated with a risk of corneal ectasia, a severe complication of refractive surgery [28]. Meanwhile its location in the cornea might be an indicator of the location of corneal ectasia progression [29]. Thus, measuring the thinnest point in the cornea prior to performing refractive surgery could be of great help to surgeons.

In addition, a 10-μm increase in CCT measured by FD-OCT was linked to an increase in IOP by 0.37 mm Hg via noncontact tonometry, similar to the effect reported in a study by Wei and associates of Chinese juveniles (10 μm CCT/0.32mmHg IOP) and in a study by Zhang and associates in Chinese adults (10 μm CCT/0.30mmHg IOP) [9,10]. However, this proportion varied greatly between studies depending on the instruments used to measure CCT and IOP. Consistent with previous studies, we did not observe an association between CCR and IOP measured by non-contact tonometry. CCR might influence the IOP measured by Goldmann applanation tonometry, but not by non-contact tonometry [30,31]. There is no existing literature describing CCT measured by FD-OCT in school-aged children, and our study provides a valuable reference for the diagnosis of glaucoma in Chinese children.

We found that in the inferior temporal and the inferior regions (both 2 to 5 mm and 6-mm diameters), CT was inversely associated with age; however, in the other parts of the cornea, including the center, this relationship was not observed. Hussein and associates reported that the CCT and CT 3 mm from the center increased gradually in pediatric subjects and reached adult thickness at approximately 5 to 9 years of age [21]. The Pediatric Eye Disease Investigator Group also reported that CCT increased until subjects reached 11 years of age [12]. Likely because the children included in the present study were older, we did not observe this increase with age. In an older cohort of Chinese school children 7 to 18 years old, Wei and associates did not find a relationship between age and CCT [10]. Zheng and associates also reported a non-significant association between age and CCT or CT 3 mm from the pupil center in Chinese children 8 to 16 years old [9]. Wang and associates found that the CT in the 4 to 7 mm and 7 to 10-mm diameter regions but not the CCT was negatively associated with age in Chinese people ranging from 12 to 89 years old [23], in line with the data of the present study. A negative association between age and para-central or peripheral CT but not CCT has also been reported in western countries [32–34].

The association between gender and CT remains controversial in both children and adults [8, 9, 10, 12, 24, 35–37]. Usually male subjects are considered to have a thicker CT than females. In the present study, gender was found to be associated with CT in most parts of the cornea but not in the central, inferior nasal, superior nasal or nasal areas. However, the association between CCT and gender was marginal (p = 0.075), indicating a possible relationship.

Refraction is another associated factor reported in several studies of both children and adults. People with increased myopic refraction were reported to have a reduced CCT compared to those with greater hyperopic refraction [10, 24, 36, 38]. However, in a study of 5,158 normal subjects, no correlation was found between refraction and CCT [39]. The Correction of Myopia Evaluation Trial (COMET) found that CCT was associated with vitreous chamber depth but not refractive error [40]. Ortiz and associates found that CCT and CT 3 mm from the apex were similar among different degrees of myopia [41]. A lack of association between CCT and refraction was also observed in a Chinese myopic population of 714 adults [42]. In addition, in studies measuring CCR (or corneal power) and axial length, CCR and axial length, but not refraction, were correlated with CCT [37,43]. In a study by Nangia and associates, after the exclusion of eyes with a corneal refractive power of over 45 diopters, the relationship between axial length and CCT became insignificant; however, the association between corneal refractive power (the reciprocal of CCR) and CCT remained [37]. In the present study, CCR was positively correlated with the minimum, central, temporal, inferior temporal, inferior, inferior nasal, and nasal areas of the cornea by multiple regression analyses. Shimmyo and associates also demonstrated a significant correlation between CCT and CCR in Caucasians, Asians, Hispanics, and African Americans [31]. In Singaporean schoolchildren, a significant association between CCT and CCR was observed, but no correlation was found between CCT and axial length [8].

Like most studies, the present study has limitations. First, only two schools in Shanghai were included, which might influence the generalizability of the study. The sample size was relatively small when the means for each age interval were determined; however, it was qualified according to statistics [15]. Second, the CT profiles presented in our study covered a 6-mm diameter region of the cornea, which might not be sufficient to diagnose peripheral cornea diseases or to assess the effect of treatment. For the diagnosis of corneal diseases that infringe upon more peripheral parts of the cornea (i.e., marginal degeneration), thickness profiles that cover a larger area are needed. However, a 6-mm diameter might be sufficient to plan refractive surgery and to diagnose keratoconus [44]. Third, we generated CT maps using FD-OCT, which might impede the comparison of our results with those of other studies. Compared with ultrasound pachymetry, which is the gold standard for measuring corneal thickness, the CT values measured by FD-OCT were smaller [14,19]. However, this might not influence the exploration of the associated factors of CT because any errors were systematic for every subject measured. In addition, as with most studies, the present study provided the position and thickness of the thinnest points of the cornea but not the thickest points because information regarding the thinnest points is more valuable to surgeons for selecting proper candidates for refractive surgery and when designing the surgery. Finally, we measured axial length and CCR after cycloplegia, which might influence the accuracy of the measurement [45,46]. However, the changes in axial length and corneal curvature radius after cycloplegia might not influence the results of the regression analyses performed in the present study because the differences were too small to be clinically relevant [47].

In summary, the present study provided corneal thickness profiles, including CCT, para-central CT, peripheral CT and the minimum CT, for Chinese school-aged children using FD-OCT. In the center, CT was only associated with CCR, but in the para-central and peripheral areas, CT was associated with younger age, male gender, and a flatter cornea.

## Supporting Information

S1 Clinical Checklist For PLOS ONE clinical study. (DOC)

S1 STROBE Checklist For cross-sectional clinical study. (DOC)
